# Response of rhizosphere bacterial community of *Taxus chinensis* var. *mairei* to temperature changes

**DOI:** 10.1371/journal.pone.0226500

**Published:** 2019-12-12

**Authors:** Xianghua Yu, Xinxing Liu, Xueduan Liu

**Affiliations:** 1 School of Minerals Processing and Bioengineering, Central South University, Changsha, China; 2 Key Laboratory of Biometallurgy of Ministry of Education, Central South University, Changsha, China; 3 Key Laboratory of Hunan Province for Comprehensive Utilization of Superiority Plant Resources in Southern Hunan, Hunan University of Science and Engineering, Yongzhou, Hunan, China; Purdue University, UNITED STATES

## Abstract

**Background:**

Temperature is a key factor influencing the growth and distribution of *Taxus chinensis* var. *mairei*, which is of high medicinal value. However, there is little information about the changes in rhizosphere bacterial community of *Taxus chinensis var*. *maire* under different temperatures.

**Methods:**

In this study, the rhizosphere bacterial communities of *Taxus chinensis var*. *maire* under a series of temperatures [5°C (T5), 15°C (T15), 25°C (T25), 35°C (T35)] were assessed through high-throughput sequencing. And some taxa annotated as Mitochondria were positively correlated with the activity of SOD.

**Results:**

Activity of peroxidase (POD) and superoxide dismutase (SOD) were increased and decreased respectively with increasing incubation temperature, showing that SOD may be the dominant reactive oxygen species (ROS) detoxifying enzyme in *Taxus chinensis var*. *maire* under low temperature. *Taxus chinensis* var. *maire* enriched specific bacterial taxa in rhizosphere under different temperature, and the rhizosphere bacterial diversity decreased with increasing temperature.

**Conclusion:**

The results indicated that rhizosphere bacteria may play important role for *Taxus chinensis var*. *maire* in coping with temperature changes, and the management of rhizosphere bacteria in a potential way to increase the cold resistance of *Taxus chinensis* var. *mairei*, thus improving its growth under low temperature and enlarging its habitats.

## Introduction

*Taxus chinensis var*. *mairei* is an important medicinal plant species, because multiple bioactive compounds with various pharmacological activities have been isolated from it [1–2]. It is an endemic species mainly distributed in central and southern China. In recent years, it is endangered in China, and the natural forests with *Taxus chinensis var*. *mairei* are only found in Guangxi province and the southeastern area of Shanxi province [3]. It is the need of the hour to protect and enlarge its habitats. However, low temperature is a great limiting factor for *Taxus chinensis var*. *mairei* growth, making it difficult for *Taxus chinensis* var. *mairei* to grow in areas with low temperature, especially in the north of Qinling Mountains in China.

Temperature is a key factor influencing plant distribution and growth [4–5]. Low temperature has many damaging effects on plants, such as disturbing the stability of proteins, increasing the accumulation of reactive oxygen species (ROS), and affecting gene and protein expression [5] Malondialdehyde (MDA), a byproduct of enzymatic and oxygen radical-induced lipid peroxidation, is widely used as a biomarker to evaluate oxidative stress in plants [6]. Some plants have evolved several mechanisms to deal with damages by regulating protein synthesis, controlling membrane composition changes, and activating active oxygen scavenging systems. Peroxidase (POD) and superoxide dismutase (SOD) are two antioxidant enzymes widely distributed in plant cells, which can eliminate ROS [7–8], and these two enzymes are also considered as sensors to prevent the occurrence of oxidative stress. Plants can achieve low-temperature resistance by acclimation, called “cold acclimation” [9]. In general, cold acclimation consisting of 1, 2, or 3 stages requires days or weeks for full development [10]. And many studies have been conducted to reveal the mechanisms of cold acclimation, and it has been revealed that the C-repeat binding factor (CBF) cold response pathway is closely related with cold acclimation [11–13]. However, most of the results were based on the model organism, *Arabidopsis thaliana* [14–15], and there is little information about the cold acclimation of *Taxus chinensis* var. *mairei*.

Soil microbe is a vital component in ecosystems, and is greatly impacted by temperature [16]. Wu et al [17]found that contrasting temperatures directly affect the diversity and structure of soil bacterial community, and warming significantly accelerates the temporal turnover of soil bacterial community. In addition, they also revealed the significant correlations between soil bacterial communities and the activity of phosphatase and urease, indicating the changes of bacterial communities were coupled with the changes of functions. Low temperature is also a key factor limiting microbial growth. However, microbial species have developed some strategies to adapt to low temperature, including changes in membrane composition and in the translation and transcription machineries [18]. Koyama et al [19] have found that the response of soil bacterial community to temperature changes was mediated by plants, indicating the close interaction and co-variation between bacterial community and plants. Studies have also found that some plant growth-promoting rhizobacteria (PGPR) could enhance the resistance of plant to cold stress [20], and cold-tolerant PGPRs can be used as bioinoculants for cold stress management [21]. Ait et al. [22] found that the chilling resistance of grapevine plantlets was enhanced by inoculating with a PGPR, *Burkholderia phytofirmans* strain PsJN, which correlated with the increase in root growth, plantlet biomass, starch, proline, and phenolics. Mishra et al.[21] revealed that inoculation with psychrotolerant Pseudomonads could alleviate cold stress of wheat (*Triticum aestivum L*.). The mechanisms of cold resistance enhancement by PGPRs include enhanced physiological condition, membrane modifications, enhanced root proliferation, increased metabolite regulation, enhanced pigmentation and increased ROS detoxifying substances [23–25].

Rhizosphere is an environment where plants interact with other organisms. It constitutes the first plant-influenced habitat encountered by soil microbes [26], and it is considered that plant development, growth, and tolerance to abiotic stress (including low temperature) have benefited from rhizosphere microbes [27–28]. It has been found that some bacteria in soil could reduce membrane damage and increase the activity of antioxidant enzymes in leaf [29]. However, there is little information about changes in microbial communities in the rhizosphere of *Taxus chinensis* var. *mairei* under different temperatures, and correlations between the changes of rhizosphere microbial communities and *Taxus chinensis var*. *mairei* growth is also little known. In this study, rhizosphere bacterial community was assessed through high-throughput sequencing, and the aim of this work was to reveal the response of rhizosphere bacterial community of *Taxus chinensis* var. *mairei* to temperature changes, and correlations between rhizosphere bacterial community and ROS detoxifying substances in *Taxus chinensis var*. *mairei*.

## Materials and methods

### Experimental design

The plants used in this study are 3 years old *Taxus chinensis var*. *mairei*. Before the experiment, plants were incubated under natural conditions for 20 days (23–25 °C, watered every 3 days). After the preculture, the plants were cultivated in plant growth chamber under four temperatures (treatments), 5°C (T5), 15°C (T15), 25°C (T25), 35°C (T35) with 3 replicates for each temperature. The relative humidity of the chamber was 80%. All the plants experienced 12 hours of light and 12 hours of dark a day, and the leaf photosynthetically active radiation was 250–350 μ mol·m^-2^·s^-1^.

### Sample collection, ROS detoxifying enzymes, and MDA measurement

Leaf samples were collected at 1, 2, 4, 8, 12, 24, 48, 72, 168 h for ROS detoxifying enzymes and malondialdehyde (MDA) measurement. 5 mature leaves were collected from each plant and weighed. All the leaves were washed three times using distilled water in the same temperature as that in the incubation chamber. Then residual moisture on the surface was dried. The leaves were then homogenized for ROS detoxifying enzymes and MDA measurement. The activity of peroxidase (POD), superoxide dismutase (SOD), catalase (CAT), and the content of malondialdehyde (MDA) were measured using relevant kits (Nanjing Jiancheng Bioengineering Institute, Nanjing, China) according to the user's manual.

The rhizosphere soil samples were collected according to the method described by McPherson et al. [30]. Briefly, after taking out the plants from the soil, shook the roots to remove bulk soil. Then the roots were suspended in a 50 mL tube containing 35 mL sterile phosphate buffer (pH = 6.5). Shook the tubes for 2 min to release the rhizosphere soil. The tubes were then centrifuged, and the supernatant was removed. The soil sediment was used for bacterial community analysis.

### Soil DNA extraction, PCR, and high-throughput sequencing

Soil total DNA was extracted from 0.5 g soil using FastR DNA SPIN Kit (MP Biomedicals, Santa Ana, CA, United States) following the protocol described in the manual.

PCR and high-throughput sequencing were performed according to previous study[31]. In brief, primer set 515F/806R were used to amplify the V4 region of the bacterial 16S rRNA gene. After purifying with AMPure XP beads (Beckman Coulter Inc., Brea, CA, United States), the PCR products were sequenced on a Illumina HiSeq 2500 system. The raw data were deposited in European Nucleotide Archive (Accession No. PRJEB32579).

### Analysis of the high-throughput sequencing data

Bioinformatic analysis of the high-throughput sequencing data was performed using Quantitative Insights Into Microbial Ecology (QIIME) (version 1.9.1) [32]according to previous study[31]. The forward and reverse reads were joined with a minimum overlap of 10 bp and maximum allowed 10% mismatches within the overlap region. Low-quality sequences (Phred quality score < 20 or length shorter than 200 bp) were discarded. Chimeras were removed using UCHIME algorithm in USEARCH package (version 10.0.240) [33] against the “Gold” database. The clean reads were then clustered into operational taxonomic units (OTUs) at 97% similarity using UCLUST method [34]. The most abundant sequence within each OTU was selected as the representative sequence. Taxonomy of each OTU was assigned using RDP Classifier [35] based on the silva 16S database (version 132). Singletons and OTUs not assigned as bacteria (including chloroplast) were removed. The OTU table was normalized by rarefaction to 24000 sequences per sample for statistical analysis.

### Statistical analyses

Principal coordinates analysis (PCoA) was performed based on Bray-Curtis distance in R (version 3.5.3, vegan package) to analyze bacterial beta diversity in different treatments. Faith’s index and Chao 1 index were calculated to compare bacterial phylogenetic diversity and richness [36]. Correlations between bacterial community and ROS detoxifying enzymes activity, and MDA content were estimated using Mantel test in R (vegan package), the dissimilarity matrices of bacterial community are represented by Bray-Curtis distance, and the dissimilarity matrices of POD, SOD, CAT, and MDA are represented by Euclidean distance. Indicator analysis was done using labdsv package in R.

## Results

### Changes of ROS detoxifying enzymes and MDA during cold acclimation

**Fig 1:**
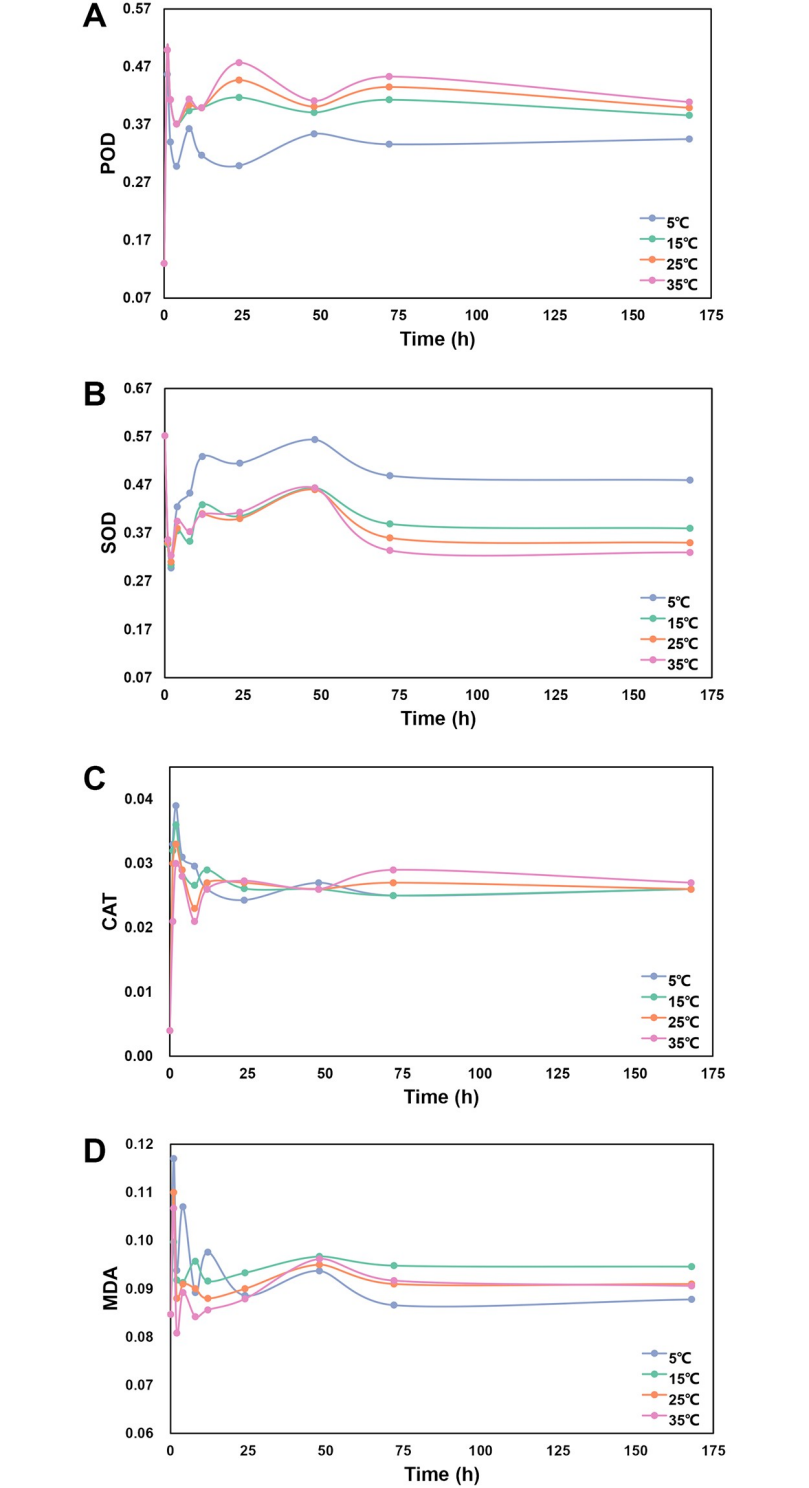
ROS detoxifying enzymes activity and MDA content. (A) Changes of POD activity during incubation time. (B) Changes of SOD activity during incubation time. (C) Changes of CAT activity during incubation time. (D) Changes of MDA content during incubation time.

Three enzymes (POD, SOD, and CAT) for ROS detoxifying and MDA were measured during the cold acclimation (Fig 1). The three enzymes and MDA varied greatly at the beginning of the incubation. After 12 h, they tended to be stable. The activity of POD increased with increasing incubation temperature (Fig 1A, S1 Table), while that of SOD were opposite, which were higher in the plants incubated at lower temperature (Fig 1B). However, the activities of CAT varied little among treatments (Fig 1C). The content of MDA was the highest in the plants incubated in the temperature of 15°C, and the lowest was observed under 5°C (Fig 1D).

### Changes of bacterial community under different temperature

**Fig 2:**
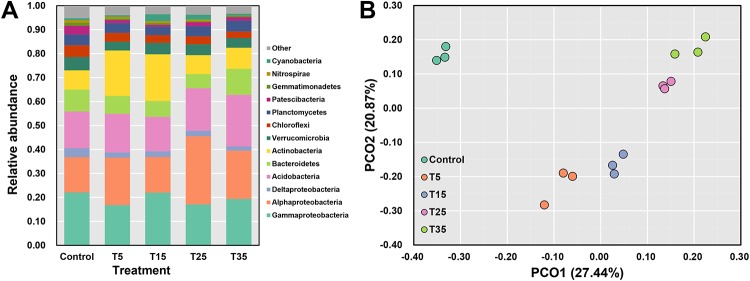
Bacterial community. (A) Rhizosphere bacterial community composition under different temperature. (B) PCoA ordinations of the Rhizosphere bacterial community based on the Bray–Curtis distance.

After quality filtering, 467041 high-quality sequences were obtained. The bacterial communities were dominated by 12 phyla including Proteobacteria, Acidobacteria, Bacteroidetes, Actinobacteria, Verrucomicrobia, Chloroflexi, Planctomycetes, Patescibacteria, Gemmatimonadetes, Nitrospirae, and Cyanobacteria (Fig 2A).

The PCoA plot based on Bray-Curtis distance showed the changes in bacterial communities under different treatments (Fig 2B). The first two axes accounted for nearly half of the community. The original soil was obviously separated from the incubated soils. And the treatments under different temperature were separated from each other. The treatment under the temperature from low to high distributed along the diagonal of the PCoA plot, indicating different temperature shaped different bacterial community in *Taxus chinensis var*. *mairei* rhizosphere soil.

Indicator species were determined to identify the specific OTUs associated with the different treatment (Table 1). Results showed that *Taxus chinensis* var. *mairei* enriched specific bacterial taxa under different temperature.

**Table 1:** Indicator species by treatment regime.

Cluster	OTU ID	Indicator value	Probability	Relative abundance (%)				Taxonomy^†^
				T5	T15	T25	T35	
T5	OTU19664	0.4625	0.017	1.44	0.86	0.37	0.44	D4_Micrococcaceae
	OTU70695	0.5431	0.018	0.29	0.10	0.08	0.07	D4_Sphingobacteriaceae
	OTU59314	0.5535	0.030	0.25	0.12	0.03	0.06	D5_*Mucilaginibacter*
	OTU117092	0.4125	0.030	0.24	0.15	0.07	0.12	D5_*Sinomonas*
	OTU80337	0.3514	0.016	0.17	0.11	0.12	0.09	D4_Nitrosomonadaceae
	OTU67569	0.4709	0.019	0.12	0.06	0.04	0.04	D4_Microscillaceae
	OTU706	0.5409	0.020	0.12	0.04	0.02	0.04	D4_Microscillaceae
	OTU8506	0.6176	0.026	0.09	0.01	0.02	0.02	D4_Gemmataceae
	OTU31704	0.6364	0.021	0.08	0.01	0.02	0.02	D4_Isosphaeraceae
	OTU84282	0.7941	0.015	0.08	0.01	0.01	0.00	D4_Microscillaceae
T15	OTU79563	0.6770	0.018	0.13	0.61	0.08	0.08	D6_*Paraburkholderia tropica*
	OTU61426	0.5020	0.036	0.34	0.53	0.15	0.03	D5_*Flavisolibacter*
	OTU65982	0.6024	0.022	0.12	0.34	0.04	0.06	D5_*Massilia*
	OTU103257	0.4771	0.045	0.07	0.14	0.05	0.04	D4_Steroidobacteraceae
	OTU23249	0.6781	0.028	0.01	0.14	0.02	0.04	D3_Bacillales
	OTU70670	0.4624	0.018	0.08	0.12	0.04	0.02	D4_Chitinophagaceae
	OTU18340	0.5364	0.019	0.02	0.08	0.03	0.02	D6_*Fusarium solani*
	OTU111993	0.6203	0.037	0.02	0.07	0.02	0.01	D5_*Flavisolibacter*
	OTU9219	0.5972	0.034	0.01	0.06	0.01	0.03	D4_Micromonosporaceae
	OTU4817	0.4938	0.049	0.04	0.06	0.01	0.00	D5_*Segetibacter*
T25	OTU49582	0.5491	0.02	0.11	0.01	0.4	0.2	D5_*Bordetella*
	OTU95945	0.3448	0.03	0.23	0.2	0.29	0.12	D5_Ellin6067 (Nitrosomonadaceae)
	OTU23598	0.4598	0.027	0.07	0.11	0.29	0.16	D3_Tepidisphaerales
	OTU120261	0.3854	0.021	0.08	0.10	0.17	0.09	D2_Gammaproteobacteria
	OTU9883	0.5989	0.017	0.03	0.03	0.16	0.04	D3_Tepidisphaerales
	OTU61986	0.4607	0.047	0.05	0.03	0.11	0.05	D1_Elusimicrobia
	OTU10059	0.4211	0.017	0.07	0.05	0.11	0.04	D5_*Chitinimonas*
	OTU53828	0.3757	0.047	0.06	0.05	0.10	0.06	D5_*Bauldia*
	OTU1228	0.3829	0.026	0.05	0.05	0.09	0.05	D4_Beijerinckiaceae
	OTU8979	0.4634	0.036	0.02	0.03	0.08	0.04	D4_Pedosphaeraceae
T35	OTU27184	0.6735	0.030	0.67	0.22	1.32	4.55	D4_Chitinophagaceae
	OTU57655	0.3443	0.015	1.30	1.34	1.66	2.26	D3_Acidobacteriia Subgroup 2
	OTU55291	0.3245	0.020	0.44	0.38	0.56	0.66	D5_*Acidibacter*
	OTU13681	0.6072	0.018	0.13	0.03	0.12	0.44	D5_*Mucilaginibacter*
	OTU98942	1.0000	0.018	0.00	0.00	0.00	0.44	D4_PHOS-HE36
	OTU72388	0.5037	0.019	0.13	0.11	0.14	0.38	D4_Acidobacteriaceae Subgroup 1
	OTU20674	0.4662	0.015	0.11	0.11	0.12	0.30	D4_Rhodanobacteraceae
	OTU46052	1.0000	0.016	0.00	0.00	0.00	0.26	D5_*Nitrosomonas*
	OTU51404	0.8394	0.022	0.01	0.01	0.03	0.25	D5_*Granulicella*
	OTU121134	0.9928	0.013	0.00	0.00	0.00	0.19	D5_*Sulfurifustis*

Only the top ten indicators in relative abundance were shown.

^†^ Classification level: D1, phylum; D2, class; D3, order; D4, family; D5 genus; D6, species.

### Changes of bacterial α-diversity under different treatment

**Fig 3:**
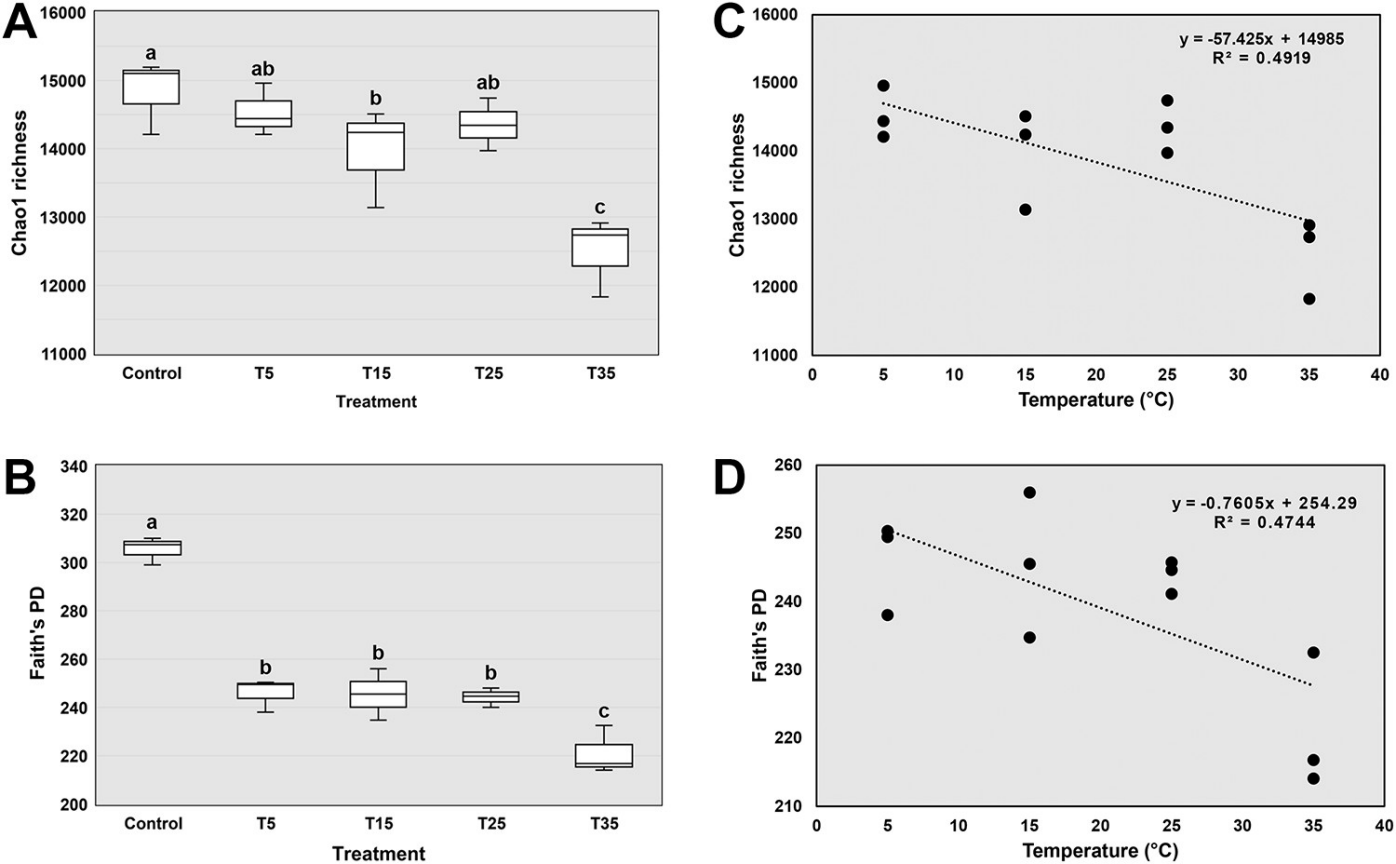
Bacterial diversities and their correlation with temperature. (A) Chao1 richness of rhizosphere bacterial communities. (B) Faith’s phylogenetic diversity of rhizosphere bacterial communities. Same alphabets above the box indicate no significant difference between treatments (detected by Kruskal-Wallis test at 0.05 level). (C) The correlation between temperature and Chao1 richness. (D) The correlation between temperature and Faith’s phylogenetic diversity.

Chao 1 and Faith’s index were used to estimate the richness and phylogenetic diversity of the bacterial communities, respectively. Highest bacterial diversity was observed in the Control soil. Soils under different treatments had lower diversity than Control, and the lowest diversity was observed in the T35 soil (Fig 3A and 3B). In addition, bacterial diversity showed significant negative correlation with incubation temperature as analyzed by linear regression (*P*<0.05) (Fig 3C and 3D).

### Correlation analysis of bacterial communities against POD, SOD, CAT, and MDA

The Spearman’s correlation coefficient determined by Mantel test showed that there were no significant correlations between bacterial community and POD, SOD, CAT, and MDA levels (S2 Table). However, the relative abundance of some OTUs significantly correlated with POD and SOD activities (Table 2).

**Table 2:** Spearman correlations between dominant OTUs and POD, SOD.

OTU ID	Enzymes	r	P value	Taxonomy^†^
OTU100240	POD	-0.748	5.12E-03	D5_*Ramlibacter*
OTU113173	POD	-0.804	1.61E-03	D4_Micrococcaceae
OTU19664	POD	-0.818	1.14E-03	D4_Micrococcaceae
OTU23328	POD	0.601	3.86E-02	D4_Microbacteriaceae
OTU4943	POD	-0.630	2.80E-02	D2_Gammaproteobacteria
OTU53448	POD	-0.634	2.68E-02	D4_Pedosphaeraceae
OTU55291	POD	0.629	2.83E-02	D5_*Acidibacter*
OTU61426	POD	-0.578	4.90E-02	D5_*Flavisolibacter*
OTU74114	POD	-0.635	2.65E-02	D4_Burkholderiaceae
OTU94713	POD	0.678	1.53E-02	D3_Subgroup 2 (Acidobacteriia)
OTU57655	SOD	-0.601	3.86E-02	D3_Subgroup 2 (Acidobacteriia)
OTU77746	SOD	-0.657	2.02E-02	D4_Mitochondria
OTU94713	SOD	-0.587	4.46E-02	D3_Subgroup 2 (Acidobacteriia)
OTU97143	SOD	0.734	6.54E-03	D4_Mitochondria

^†^ Classification level: D1, phylum; D2, class; D3, order; D4, family; D5 genus.

## Discussion

Cold stress is one of the environmental stresses limiting plant growth, productivity, and distribution [9]. The accumulation of ROS is closely associated with cold stress, which deleteriously affects plant tissues, especially the membranes [5]. ROS detoxifying enzymes, including POD, SOD, and CAT, are ubiquitous in plant cell to eliminate the ROS. In this study, the activity of SOD increased under cold stress, and it positively correlated with incubation temperature (Fig 1B), indicating more O_2_^-^ was produced under lower temperature. However, the activity of POD showed an opposite trend against SOD, and the activity of CAT varied little under different incubation temperatures. This is different from a previous study which indicated that increase in CAT activity was the highest among the three ROS scavengers (SOD, CAT, and POD) under cold temperature. This difference may be due to high abundance of H_2_O_2_ than other ROS, as other ROS should firstly be converted to H_2_O_2_ for further deoxidation [37]. However, the conversion of H_2_O_2_ could be catalyzed by multiple types of antioxidant enzymes, such as ascorbate peroxidase (APX) and glutathione peroxidase (GPX)[7], besides POD and CAT. Thus, we speculate that the oxidative stress caused by O_2_^-^ may play an important role in influencing plant growth under low temperature.

Rhizosphere, the narrow zone between soil particles and plant roots, is of paramount importance for ecosystem services. It contains various types of microbes, including bacteria, fungi, and other microeukaryotes, and is the most active area for biogeochemical cycling [25]. The plant-microbe interaction in the rhizosphere region is closely associated with plant resistance to abiotic stress [27], including cold stress. Through high-throughput sequencing, this study revealed that the rhizosphere bacterial community of *Taxus chinensis var*. *mairei* varied with temperature changes (Fig 2, Table 1), indicating that the plant may enrich specific bacterial taxa to help it alleviate cold stress and confront temperature changes. Previous study reported that the inoculation of plant growth-promoting rhizobacterium could enhance the chilling resistance of *Vitis vinifera L*. and increase grapevine growth and physiological activity at a low temperature[24]. Another study found that inoculation of tomato with psychrotolerant PGPR increased tomato chilling resistance, reduced membrane damage, and activated antioxidant enzymes by decreasing electrolyte leakage and malondialdehyde content and improving the activity of ROS scavengers, such as SOD, APX, and glutathione synthase (GSH) [29]. In this study, we found that the relative abundance of some bacterial taxa significantly correlated with the activity of POD and/or SOD, which indicated their role in the increase of cold resistance of *Taxus chinensis var*. *mairei*. It is notable that the diversity of rhizosphere bacterial community showed negative correlation with temperature, which may indicate that the dependence on bacterial community by the host plant decreased when the cold stress was eliminated.

In summary, some physiological changes in *Taxus chinensis var*. *mairei* and the varication of rhizosphere bacterial community of *Taxus chinensis var*. *mairei* under serial incubation temperature were revealed. Results indicated that the activity of POD and SOD were increased and decreased respectively with increasing incubation temperature. Different temperatures shaped distinct rhizosphere bacterial communities, and some of the taxa were significantly correlated with the activity of POD and SOD, indicating the important role of rhizobacteria in alleviating cold stress of *Taxus chinensis var*. *mairei*. These results also suggest great potential of rhizobacteria in improving the cold tolerance of host plants.

## Supporting information

S1 Table The activity of ROS detoxifying enzymes and the content of MDA. (DOCX)

S2 Table Spearman’s correlation coefficient between bacterial community and POD, SOD, CAT, and MDA determined by Mantel test. (DOCX)
