# Supplementary material for: Response of rhizosphere bacterial community of Taxus chinensis var. mairei to temperature changes
Source: PLoS One. 2019 Dec 12;14(12):e0226500. doi: 10.1371/journal.pone.0226500 (PMC6907812; doi:10.1371/journal.pone.0226500)
Supplement: S1 Table — (DOCX) [file pone.0226500.s001.docx]

**S1 Table. The activity of ROS detoxifying enzymes and the content of MDA**

|  | T5 | T15 | T25 | T35 |
| --- | --- | --- | --- | --- |
| POD | 0.336±0.02c^†^ | 0.400±0.011b | 0.414±0.010ab | 0.427±0.016a |
| SOD | 0.505±0.033a | 0.403±0.033b | 0.396±0.036bc | 0.387±0.014c |
| CAT | 0.026±0.002a | 0.026±0.001a | 0.026±0.001a | 0.026±0.002a |
| MDA | 0.091±0.004a | 0.094±0.002a | 0.091±0.002a | 0.089±0.004a |

The average and standard deviation of the data after 12 h.

^†^Means followed by the same letter are not significantly different detected by Kruskal-Wallis test (*P*>0.05).
