# Supplementary material for: Response of rhizosphere bacterial community of Taxus chinensis var. mairei to temperature changes
Source: PLoS One. 2019 Dec 12;14(12):e0226500. doi: 10.1371/journal.pone.0226500 (PMC6907812; doi:10.1371/journal.pone.0226500)
Supplement: S2 Table — (DOCX) [file pone.0226500.s002.docx]

**S2 Table. Spearman’s correlation coefficient between bacterial community and POD, SOD, CAT, and MDA determined by Mantel test**

| Factors | r | p value |
| --- | --- | --- |
| POD | 0.007098 | 0.396 |
| SOD | -0.09784 | 0.685 |
| CAT | -0.07507 | 0.687 |
| MDA | -0.11993 | 0.823 |
